# Cyberknife Radiosurgery for Synovial Sarcoma Metastasizing to the Spine: Illustrative Case Reports

**DOI:** 10.7759/cureus.37087

**Published:** 2023-04-04

**Authors:** Aroosa Zamarud, David J Park, Ghani Haider, Steven D. Chang, Antonio Meola

**Affiliations:** 1 Department of Neurosurgery, Stanford University School of Medicine, Stanford, USA; 2 Department of Neurosurgery, Stanford University School of Medicine, Palo Alto, USA

**Keywords:** sarcoma, synovial sarcoma, stereotactic radiosurgery, sarcoma spine metastases, openai, chatgpt, cyberknife

## Abstract

Synovial sarcoma (SS) is a rare and aggressive type of soft tissue sarcoma that commonly affects young adults. Metastasis in the spine is a rare complication, and the management of these lesions is challenging. Radiosurgery is an increasingly popular treatment option for spinal metastasis due to its ability to deliver high doses of radiation to the target volume with minimal exposure to surrounding healthy tissues. In this paper, we present two cases of SS with spinal metastasis that were treated with CyberKnife radiosurgery (CKRS). The first case was a 52-year-old female with a history of multiple thoracotomies and lobectomies for lung metastases, who was diagnosed with T6-T8 and T4 spinal metastasis. The second case was a 53-year-old female with Down syndrome, who was diagnosed with T12-L1 spinal metastasis. Both patients experienced an improvement in their symptoms following CKRS treatment and showed stable or decreasing lesion sizes on follow-up imaging. The progression-free survival (PFS) in the first case was 37 months and overall survival (OS) was 79 months. In the second case, the PFS was 12 months and OS was 18 months. These cases highlight the potential benefits of CKRS as a treatment option for SS with spinal metastasis and support its use in the management of this challenging condition.

## Introduction

Synovial sarcoma (SS) is a soft tissue sarcoma that tends to spread to distant sites, including the lung [1]. Spinal metastasis is a rare complication in patients with SS [2], and it can cause significant morbidity. The symptoms of SS of the spine may include pain, weakness, and numbness in the affected area, as well as difficulty with movement and coordination. The diagnosis of SS typically involves imaging tests, such as MRI or CT scans, as well as a biopsy to confirm the presence of cancer cells [1].

The management of spinal metastasis is challenging, and various treatment options have been proposed, including surgery, radiation therapy, and chemotherapy [3]. The main goal of surgery is to remove as much of the tumor as possible while preserving as much function as possible. In some cases, spinal fusion may also be necessary to stabilize the spine after surgery. Radiation therapy may be used before or after surgery to shrink the tumor or kill any remaining cancer cells [4]. Chemotherapy may also be used to kill cancer cells that have spread to other parts of the body. SS has a poor prognosis despite treatment with various modalities. Local recurrence occurs in up to 30% of patients, whereas distant metastasis is noted in 50% of the patients. Patients with metastatic disease have a poor prognosis, with a survival of fewer than two years [4,5].

Stereotactic radiosurgery (SRS) has been used as a non-invasive treatment option for different sarcomas of the spine [6,7]. This technique is especially useful in the treatment of spinal tumors, as it allows for the delivery of high doses of radiation to the tumor without damaging the surrounding spinal cord [6]. CyberKnife radiosurgery (CKRS) is a non-invasive form of radiation therapy that delivers a high dose of radiation to a small, well-defined target while sparing surrounding normal tissues [8]. This technique is rarely used to treat SS of the spine. This study aimed to report two cases of SS patients who received CKRS for spinal metastases and to evaluate its effectiveness in managing their symptoms and prolonging their survival.

## Case presentation

Methods

**Figure 1:**
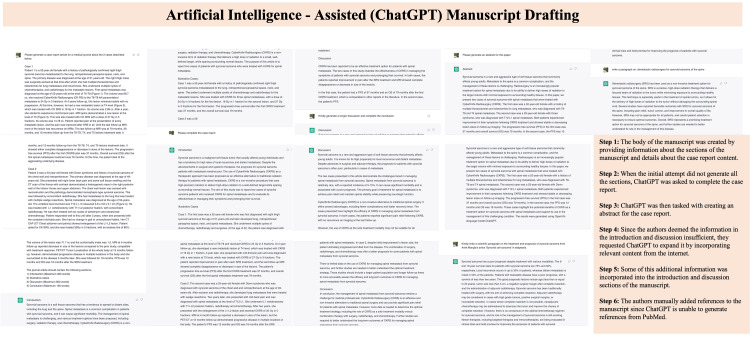
Step-by-step generation of the case report by ChatGPT

The two cases described in this article were collected from a retrospective medical record review. The information gathered included patient demographics, medical history, imaging studies, and treatment history. The data were analyzed by using OpenAI's language model, ChatGPT. ChatGPT was trained on a large corpus of text to generate case reports and has been shown to have a high level of accuracy and consistency in its outputs. Since ChatGPT does not have direct access to specific databases such as PubMed, the references were added manually by the author (Figure 1).

Illustrative cases

Case 1

**Figure 2:**
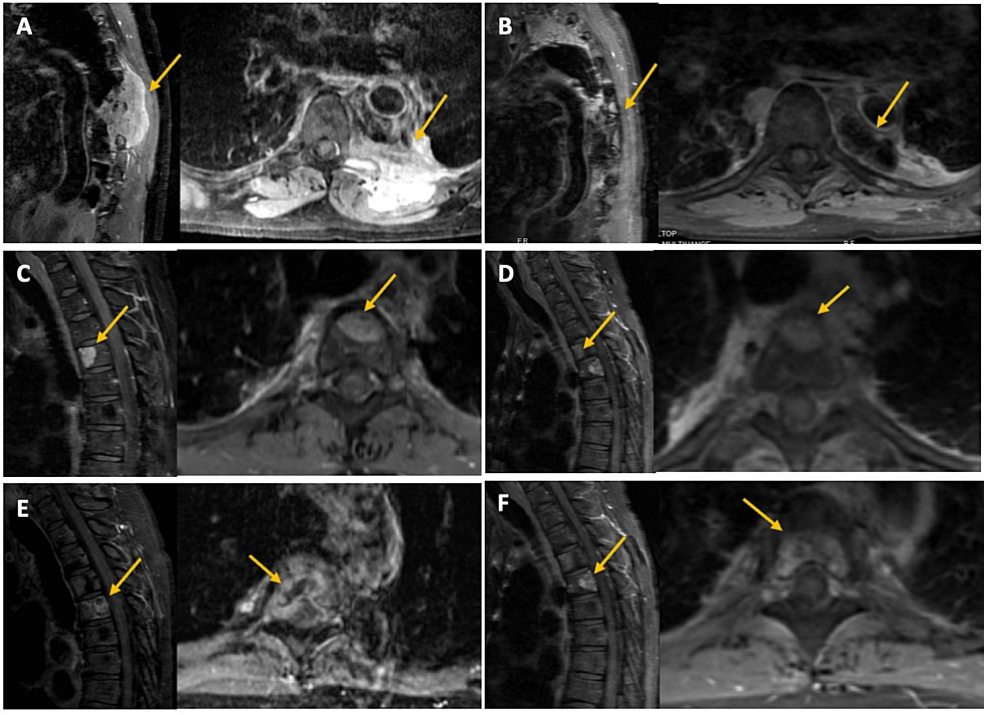
Pre- and post-CKRS scans (A) Sagittal (left) and axial (right) T1 contrast scan of T6-T8 tumor pre-treatment (B) Sagittal (left) and axial (right) T1 contrast scan of T6-T8 tumor post-treatment (48-month follow-up) (C) Sagittal (left) and axial (right) T1 contrast scan of T4 tumor pre-treatment (D) Sagittal (left) and axial (right) T1 contrast scan of T4 tumor post-treatment (12-month follow-up) (E) Sagittal (left) and axial (right) T1 contrast scan of T5 tumor pre-treatment (F) Sagittal (left) and axial (right) T1 contrast scan of T5 tumor post-treatment (5-month follow-up)

The first patient was a 52-year-old female with a history of SS that had metastasized to the lung, retroperitoneal paraspinal space, neck, and spine. The primary disease was diagnosed at the age of 31 years old and was surgically excised. After this, she had multiple thoracotomies and lobectomies for lung metastases and recurrences. She also received multiple cycles of chemotherapy and radiotherapy for the metastatic lesions. The chemotherapeutic agents used were ifosfamide, dacarbazine-doxorubicin, pazopanib, and trabectedin. The first spinal metastasis was diagnosed at the age of 52 years old at the level of T6-T8 (Figure 2-A) and was treated with CKRS with 24 Gy in three fractions. The volume treated was 68.7 cc. At the four-year follow-up, the lesion was well-controlled with no progression (Figure 2-B). However, at this time, she presented with a new metastatic lesion at the T4 level (Figure 2-C) which was treated with CKRS in 18 Gy in one fraction to a volume of 2.08 cc. After a year, she started to experience mid-thoracic pain, and an MRI of the spine showed a new lesion at the T5 level (Figure 2-E), which was also treated with CKRS in 27 Gy in three fractions to a volume of 14.78 cc. The patient reported pain improvement after every CKRS treatment, and until the last follow-up MRI, which was at 70-month, 33-month, and 12-month follow-up from the T6-T8, T4, and T5 lesion treatment date, none of the lesions had progressed (Figure 2-B, D, and E). The progression-free survival (PFS) after the first CKRS plan was 37 months (calculated from the time of treatment to the time of progression or death, whatever comes first), and the overall survival (OS) after the first spinal metastases treatment was 79 months (calculated from the time of treatment to the time of progression or death, whatever comes fist). At this time, the patient died of the aggravating underlying disease.

 

Case 2

**Figure 3:**
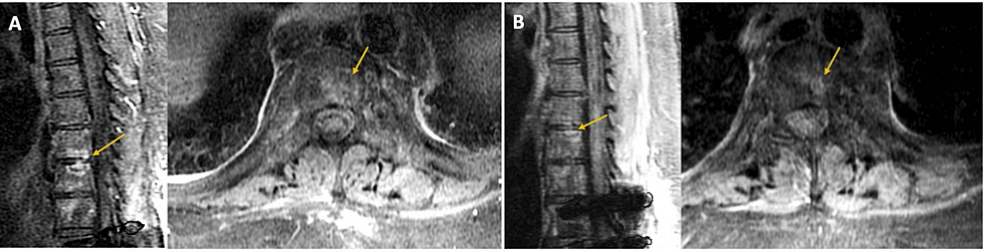
Pre- and post-CKRS T1 contrast scans (A) Sagittal (left) and axial (right) T1 contrast scan of L1-L2 tumor pre-treatment (B) Sagittal (left) and axial (right) T1 contrast scan of L1-L2 tumor post-treatment (four-month follow-up)

The second patient was a 53-year-old female with Down syndrome and a history of SS of the chest wall and retroperitoneum. The primary disease was diagnosed at the age of 49 years old, and she underwent excision of the chest wall lesion followed by radiotherapy. She had metastases to the lung that were treated with multiple wedge resections. The pathology reported monophasic-type SS. Spinal metastasis was diagnosed at the age of 53 years old. The vertebral level involved was T12-L1, and it measured 3.8 x 3.8 x 5.1 cm. She was treated with L1 vertebrectomy with T11-L2 posterior fixation, followed by six cycles of chemotherapy. The chemotherapeutic agents used were doxorubicin-olaratumab and pazopanib. After 2 years, she presented with mid-back pain, and her CT chest, abdomen, and pelvis showed enlargement of the L1-L2 lesion (Figure 3-A). She was treated with CKRS at 35Gy in five fractions to a volume of 71.7 cc. MRI at the 4-month follow-up reported a decrease in the size of the lesions (Figure 3-B), but PET/CT tumor localization of the whole body at the 12-month follow-up demonstrated progressive disease in multiple locations in the body. The patient succumbed to the disease five months later. The PFS after the SRS treatment was 12 months, and the overall survival was 18 months.

Results

In both cases, the patients had SS that had metastasized to the spine. Both patients received radiosurgery as a treatment option and showed improvement in their symptoms. In the first case, the lesion at the T6-T8 level remained stable with no progression at the four-year follow-up, and the lesion at the T4 level was treated successfully with a single fraction of CKRS. The lesion at the T5 level was treated with CKRS and showed a decrease in size according to the MRI scans at the 12-month follow-up.

In the second case, the patient was treated for a recurrent tumor at the L1-L2 level with CKRS. MRI scans at the seven-month follow-up showed a decrease in the size of the lesion, but PET/CT scans at the 12-month follow-up showed progressive disease in multiple locations in the body.

These cases illustrate the use of CKRS in treating spinal metastases from SS. Although CKRS can provide pain relief and stability of the lesions, it may not necessarily lead to prolonged survival in all cases, as shown in the second case. Further studies with larger patient populations are needed to establish the role of CKRS in the management of spinal metastases from SS.

## Discussion

SS is a rare and aggressive type of soft tissue sarcoma that primarily affects young adults [1]. It is known for its high propensity for local recurrence and distant metastasis [9]. Despite advances in surgical and adjuvant therapy, the prognosis for patients with SS is often poor, particularly in cases of metastasis [4,10].

The two cases presented in this article demonstrate the challenges faced in managing spinal metastasis from SS. Spinal metastasis from SS is relatively rare, with a reported incidence of 5-15%. According to our literature search, less than 50 cases of spinal SS are published in the literature, the majority of which are treated with surgical resection. The majority of the published papers are case reports [2]. The primary goal of treatment for spinal metastasis is to achieve pain relief and maintain spinal stability while preserving neurologic function. It can cause significant morbidity and is associated with a poor prognosis. The 5- and 10-year survival rates for SS patients are 75% and 50%, respectively, with a high risk of local recurrence and distant metastasis [4,10]. Surgery remains the mainstay of treatment, and adjuvant radiotherapy may be considered in cases with high-grade tumors, positive surgical margins, or incomplete resection. In cases where complete resection is not possible, neoadjuvant chemotherapy may be administered to downsize the tumor and improve the chance of complete resection. However, there is no consensus on the optimal chemotherapy regimen for SS, and its role in the management of SS is still evolving [10].

CKRS is a non-invasive alternative to traditional spinal surgery. It offers several advantages, including fewer complications and faster recovery times [11,12]. It is very rarely used to treat spinal SS. In a study with 29 patients, 3 patients with spinal SS were treated with SRS [13]. The cases presented here highlight the efficacy of CKRS in managing spinal metastasis from SS. In both cases, the patients reported significant pain relief following CKRS, with no recurrence on imaging at the last follow-up. However, the use of CKRS as the sole treatment modality may not be suitable for all patients with spinal metastasis. In Case 2, despite initial improvement in lesion size, the patient ultimately progressed and died from the disease. Despite the presented data, there is limited data on the use of CKRS for managing spinal metastasis from SS, and further studies are needed to better understand the optimal treatment strategy. These studies should include a larger patient population and longer follow-up times to more accurately assess the efficacy and long-term outcomes of CKRS for managing spinal metastasis from SS.

## Conclusions

The management of spinal metastasis from SS remains a challenge for medical professionals. CKRS is an effective and non-invasive alternative to traditional spinal surgery and can provide significant pain relief for patients with spinal metastasis. However, more data is needed to determine the optimal treatment strategy, including the role of CKRS as a sole treatment modality versus combination therapy with surgery, radiotherapy, and chemotherapy. Further studies are required to better understand the long-term outcomes of CKRS for managing spinal metastasis from SS.
